# Feasibility and Reliability of Grain Noise Suppression in Monitoring of Highly Scattering Materials

**DOI:** 10.1007/s10921-017-0430-8

**Published:** 2017-06-26

**Authors:** Yuan Liu, Chang Liu, Anton Van Pamel, Peter Cawley

**Affiliations:** NDE Group, Department of Mechanical Engineering, Imperial College London, Exhibition Road, London, SW7 2AZ UK

**Keywords:** Grain noise suppression, Health monitoring, Highly scattering materials, Baseline subtraction

## Abstract

A feasibility study on grain noise suppression using baseline subtraction is presented in this paper. Monitoring is usually done with permanently installed transducers but this is not always possible; here instead monitoring is conducted by carrying out repeat C-scans and the feasibility of grain noise suppression by subtracting A-scans extracted from the C-scans is investigated. The success of this technique depends on the ability to reproduce the same conditions for each scan, including a consistent stand-off, angle, and lateral position of the transducer relative to the testpiece. The significance of errors are illustrated and a 3D cross correlation is used which enables the same lateral position to be located within successive C-scans. The experimental results show that a noise reduction of around 15 dB is obtained after baseline subtraction, which will significantly improve the defect detection sensitivity. In practice however, successive C-scans may be conducted at different temperatures and with different transducers of similar specifications but a varying frequency response. Compensation techniques to reduce the impact of such variations are then presented and their effectiveness is verified experimentally. It is shown that it is feasible to obtain an overall improvement of around 10 dB in the signal to noise ratio via baseline subtraction, where a temperature difference of up to 10 $^\circ$C and a peak frequency shift of as much as ±250 kHz from a baseline value of around 7 MHz can be tolerated. However, this improvement was obtained in laboratory conditions with no changes to the surface of the specimen due to oxidation or corrosion. It is shown that differences in temperature and transducer frequency response are more difficult to compensate for than changes in test geometry and position.

## Introduction

In the next generation of power stations, high temperatures (up to 700 $^\circ$C) will be used to improve efficiency. For plants working at such high temperatures, creep deformation is of great concern, as it can weaken structures and eventually cause creep failure. Grain boundary sliding is one of the mechanisms that contribute to creep in metals [1, 2], so the creep rate is related to the grain boundary area. Thus, large grained materials are desirable as they can provide higher creep strength.

However, ultrasound inspection of large grained materials is made difficult by the presence of different alignments of principal axes, either of individual grains or colonies. This causes acoustic impedance contrasts and thus scattering from grain or colony boundaries [3]. Due to scattering, the outgoing wave is strongly attenuated as it propagates through the material and the received signals are often dominated by coherent noise. Attenuation reduces the amplitude of reflections from defects, and scattering increases the noise level, thus the resulting signal to noise ratio (SNR) is often too low for the test to be viable. In order to enhance SNR, researchers have developed many signal processing methods to suppress grain noise. The split spectrum processing[4–6] and wavelet transform de-noising methods [7–9] are widely researched techniques but show limited improvement [10]. If we assume that the ultrasonic properties of the grains are unaffected by ageing [11], grain noise is coherent and an opportunity then exists to use a structural health monitoring (SHM) approach in which a baseline reading is subtracted from the current reading, thus removing the grain noise and producing a residual signal which reveals the presence of defects. This idea has been researched extensively in guided wave health monitoring [12–14] ; this paper assesses the feasibility of applying the idea to the bulk wave ultrasonic testing of materials that generate substantial grain noise.

**Fig. 1:**
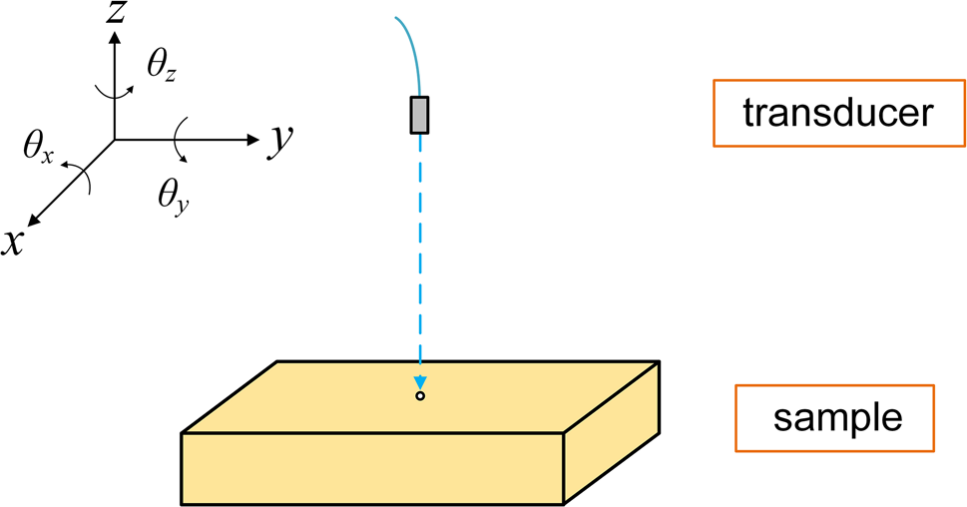
Definition of degrees of freedom in the tests

**Fig. 2:**
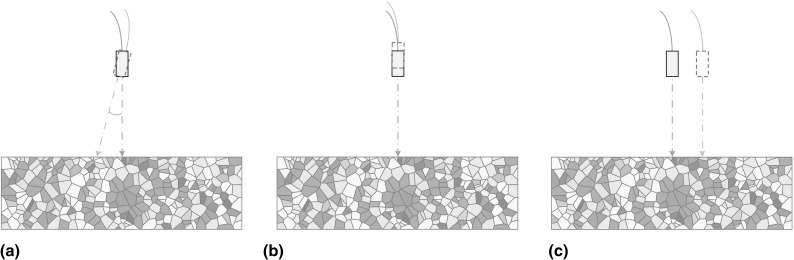
Schematic drawing (not to scale) of potential errors due to transducer setup in repeat inspections **a** different transducer angle, **b** different transducer stand-off, **c** different test point (Color figure online)

Figure 1 defines the degrees of freedom in the tests. $\theta _x$ and $\theta _y$ are the transducer angles in $y-z$ and $x-z$ planes; *z* represents the stand-off of the transducer; *x* and *y* show the test position in $x-y$ plane. Here $\theta _z$ showing rotation of the transducer in $x-y$ plane is not taken into consideration, because the transducer is axisymmetric. Figure 2 shows the situations if the relative positions of the transducer and sample are different in two tests, which will cause differences in grain noise and so reduce the effectiveness of baseline subtraction.

In addition to positional errors, another challenge is that the repeat scans may be carried out at different temperatures and with different transducers of similar but non-identical specifications. A temperature change results in a difference in wave speed, and a transducer frequency response change alters the excitation waveform. Both of these changes influence the received signals. Thus, without compensating for these effects, raw subtraction of two signals obtained in different situations can be ineffective in enhancing SNR or even make it worse. In this study, a shift and scale-transform-based stretch [15] combined method is used for temperature compensation and a frequency compensation method based on frontwall reflection spectra is developed to compensate for the transducer frequency response change.

In this paper, the feasibility and reliability of grain noise suppression in monitoring highly scattering materials using repeat scanning is studied and the success in compensating for the different test setup and environmental issues is compared. The following Sect. 2 introduces the experimental setup. Section 3 shows the reliability of locating the same position and hence generating the same grain noise in repeat scans. An existing temperature compensation method and a developed compensation method for transducer frequency response change are presented in Sect. 4. Finally, conclusions are given in Sect. 5.

## Experimental Setup

**Fig. 3:**
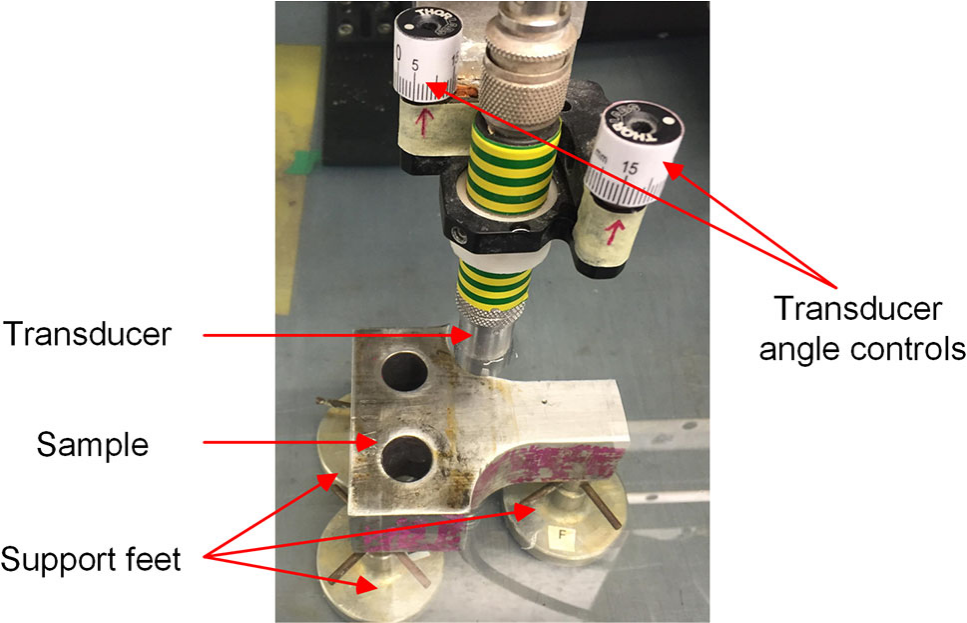
Experimental setup

The experimental setup used in the following sections is introduced here, and is shown in Fig. 3. A sample of 304 stainless steel with a thickness of 40 mm was used in all the tests. It was cut from a material testing specimen supplied by a collaborating company. The sample was placed on three support feet, as commonly used in C-scan tanks, and the transducer orientation about the $\theta _x$ and $\theta _y$ axes (defined in Fig. 1) was adjusted using calibrated controls on the transducer holder.

**Table 1:** Transducer used; all were unfocused

Transducers	Manufacturer	Centre frequency (MHz)	Diameter (in.)
1	IMASONIC	7.5	0.24
2	GE	7.5	0.50
3	GE	7.5	0.50

**Fig. 4:**
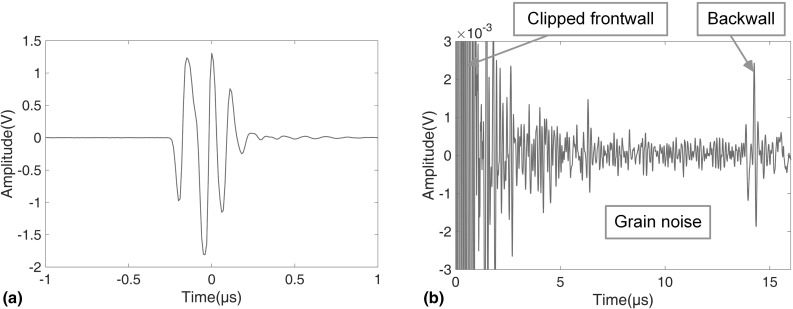
Typical A-scan signals obtained with Transducer 1 **a** frontwall, **b** whole signal with clipped frontwall

An Olympus 5077PR square wave pulser/receiver was used to send and receive the signals. In single point tests the signal was captured by a LeCroy WaveRunner 44Xi oscilloscope that has an averaging facility. The C-scans were carried out using a scanning system with an Agilent DP1400 acquisition card. The properties of the transducers used in this study are shown in Table 1. The results of Sects. 2 and 3 were obtained with Transducer 1; Transducers 2 and 3 were used in Sect. 4. It should be noted here that the purpose of this paper is to discuss the feasibility of using baseline subtraction for grain noise removal so sufficient grain noise needs to be generated in the experiments. It was found that this sample exhibits appropriate grain noise when excited by a 7.5 MHz transducer, so this centre frequency was used in all the experiments. It is not suggested that this is the optimum frequency for inspection of this component; the experimental setup was chosen simply to generate significant grain noise so that the feasibility of its removal via baseline subtraction could be investigated. Typical A-scan results are shown in Fig. 4, where to show the wave propagation time in the sample clearly, the frontwall is centred at 0s. As the intensity of the backscattered energy was significantly less than that of the frontwall reflection, the analog-to-digital converter (ADC) range used for collecting the grain noise was around 48 dB below that for frontwall capture and hence the frontwall signals are clipped in Fig. 4b.

## Repeatability Test

As shown in Fig. 2, there are several potential errors due to the transducer setup in repeat inspections that would cause the incident wave to interact with different grains and so generate different grain noise. In order to suppress grain noise with baseline subtraction, the same relative position of the transducer and sample in repeat tests must be found. In this section, the feasibility and reliability of finding this same relative position in repeat inspections is studied.

### Transducer Angle Change

It can be clearly seen that in Fig. 2a, an ultrasound wave at different incident angles interacts with different grains, hence generating different grain noise. Thus, accurately adjusting the transducer to be perpendicular to the sample in each setup is necessary. Maximum signal calibration, referred to as the standard method in this paper, is commonly used to find the normal incident angle. This method adjusts the transducer by finding a maximum amplitude of the frontwall echo. In theory, the frontwall amplitude reaches the maximum when the transducer is normal to the sample. However, around the peak, the rate of change of amplitude with angle is too small for an inspector to manually adjust the angle to a satisfactory precision.

**Fig. 5:**
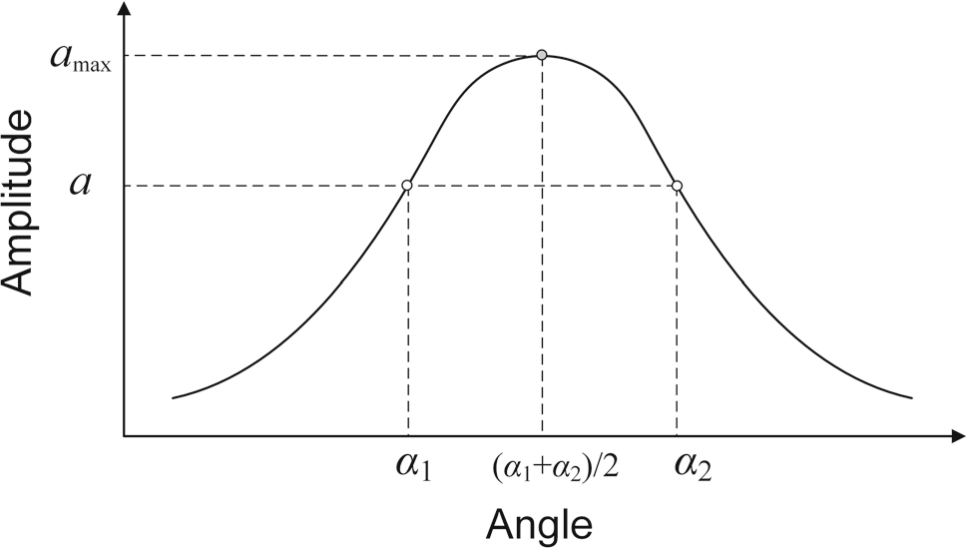
Schematic diagram of transducer alignment method

An alternative method is shown schematically in Fig. 5. Firstly, the transducer is adjusted to be roughly perpendicular to the sample using the standard method. Then the transducer is rotated about the *x* axis ($\theta _x$ in Fig. 1) until a significant amplitude reduction is seen and the amplitude and angle setting are recorded; the transducer is then rotated in the opposite direction until the same amplitude is obtained on the other side of the peak. The optimum angle is then the mean of the two recorded angles. The same procedure is then followed with the $\theta _y$ adjustment and the process can be iterated if necessary.

**Fig. 6:**
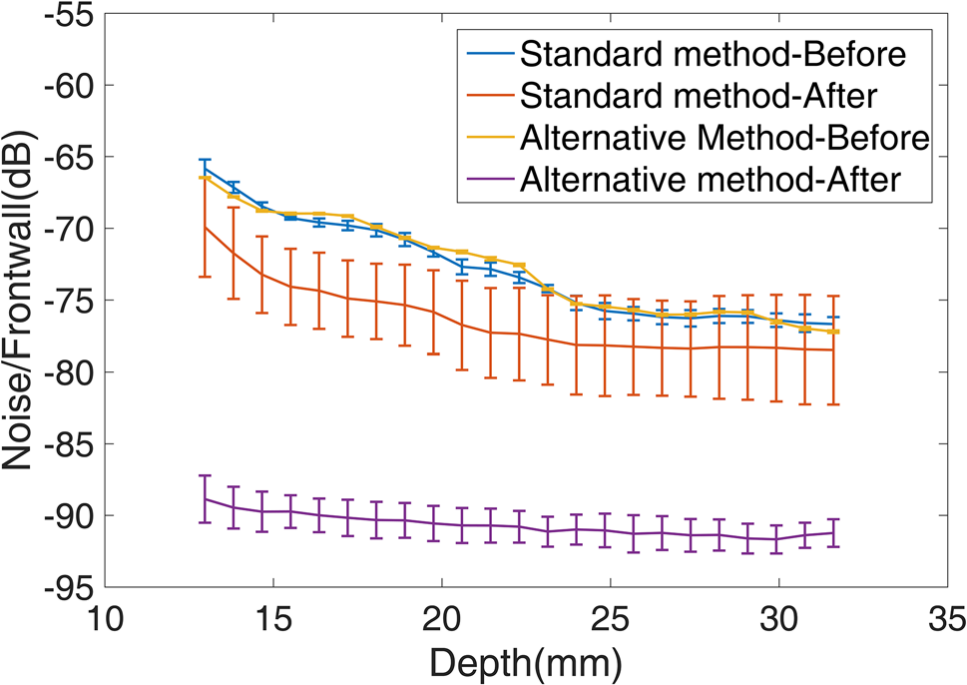
Noise to frontwall amplitude ratio versus depth before and after baseline subtraction. The data consists of eight A-scans where the first represents the baseline and the seven successive traces are used for subtraction. Each data point shows the mean (*line*) and standard deviation (*bar*) of the RMS for a 14 mm window centred about its indicated x-axis value. The range of depths considered here corresponds to the grain noise which lies in-between the frontwall and backwall signals. Electrical noise residual is around 94 dB below the frontwall signal (Color figure online)

The two calibration methods were then used to adjust the transducer angle and quantify the influence of an angle error on baseline subtraction. For each method, eight A-scans were recorded having reset the angle between each reading; the first reading was then used as the baseline and the subsequent readings were subtracted from it; the test was repeated at three randomly selected positions to verify the robustness of the method to different grain geometry. The results at one position are shown in Fig. 6; the blue and yellow curves show the mean grain noise level of the seven subsequent A-scans as a function of depth; the points show the RMS (root mean square) over a 14 mm range centred on the marked depth for the standard method and the alternative method respectively. As the RMS values are calculated over a 14 mm range, the central depth used for calculation is from 13 to 32 mm (the total depth is 40 mm) to remove the influence of the frontwall and backwall on the RMS grain noise calculation. It can be seen that there is little difference between these curves. In contrast, the red and purple curves show the mean residual level after baseline subtraction for the standard method and the alternative method respectively, together with error bars corresponding to one standard deviation of the noise level obtained from seven baseline subtracted residuals. It can be seen that the residual obtained with the alternative method, which is around 18 dB lower than the noise level in a single signal, is an average of about 14 dB better than that with the standard method and the variability is substantially lower. The level of improvement with both methods decreases with depth; this may be because when beams at different incident angles travel deeper into the sample, the spatial separation between them increases so the probability of them interacting with different grains increases. Similar results were obtained at the other two test positions. It is shown that the alternative method is more reliable for the transducer angle adjustment and it provides high repeatability and accuracy in transducer angle setting, thereby making it possible to use baseline subtraction to suppress grain noise in repeat scanning. As a cautionary note, due to a single operator performing both methods for the present investigation, the variability of operator skill is not taken into account. Although this plays a role in determining performance for both methods, we have assumed the investigator to be representative of the average skill for both methods.

**Fig. 7:**
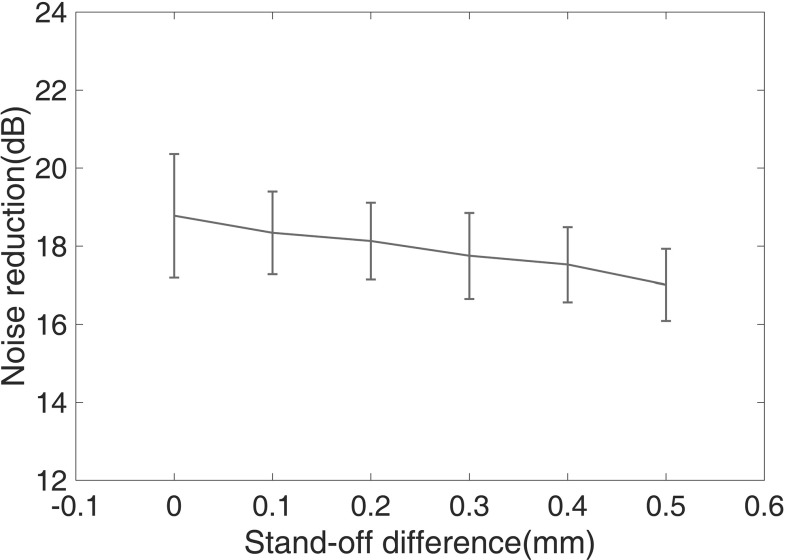
Mean noise reduction over sample depth after baseline subtraction as a function of transducer stand-off difference. *Error bars* show the standard deviation of ten repeats at each stand-off

**Fig. 8:**
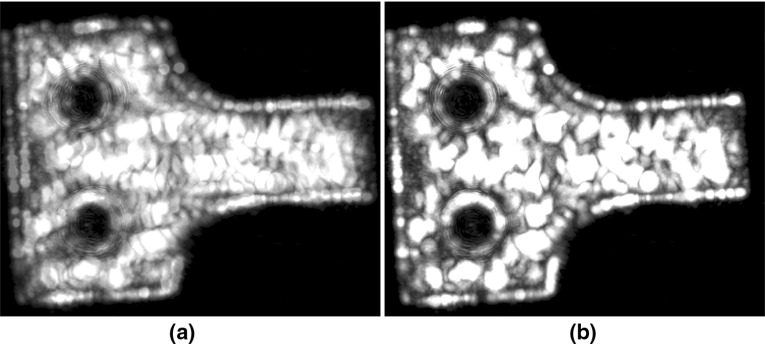
Overlapped C-scan images (backwall echo amplitude) from repeat tests **a** before and **b** after alignment

### Transducer Stand-Off

A transducer stand-off change, shown in Fig. 2b, is very likely to happen in repeat inspections. The change in the distance between the transducer and sample introduces a shift in received signals. In addition, due to beam spreading, the pressure distribution on the sample surface also changes with distance. This results in different grains interacting with the beam and so different grain noise being generated. Therefore, we need to accurately adjust the transducer stand-off to generate the same grain noise in each setup to make it possible to remove the grain noise by baseline subtraction.

Assuming a constant speed, there is a one-to-one correspondence between distance and time. Therefore, the frontwall arrival time is often used to adjust the transducer stand-off. However, in repeat tests, the temperature can be different, which causes a difference in wave speed and so changes the relationship between the distance and time. In this case, if a transducer is adjusted using the arrival time of a frontwall signal collected at a different temperature, a stand-off error will be introduced. For example, suppose a reference frontwall arrival time obtained at 19 $^\circ$C when the stand-off is 65 mm. If this reference time is used to adjust the transducer stand-off when the temperature changes to 22 $^\circ$C, a stand-off difference of around 0.4 mm will be introduced.

In order to see how much this difference influences baseline subtraction, the stand-off was changed manually from 65 to 65.5 mm to simulate the effect of a small temperature change on the transducer stand-off adjustment. At each stand-off, tests were repeated ten times. The first reading of the ten at 65 mm stand-off was used as the baseline and subsequent readings were subtracted from it. Figure 7 shows the average improvement between raw grain noise level and residual after baseline subtraction over the thickness of the sample at different stand-offs. It is clear that small variations in stand-off have only a minor effect on the residual after subtraction and the standard deviation.

### Test Position

It is obvious that if tests are carried out at different positions on the sample, the ultrasound wave will interact with different grains (as shown in Fig. 2c) and then different grain noise will be generated. If the grain noise in the baseline is different from that in the current signal, baseline subtraction will be ineffective in grain noise suppression. Therefore, in order to suppress grain noise, the subtraction needs to be conducted at the same positions. Locating a specific position by conducting single A-scans is difficult. However, it becomes possible to locate a position by aligning different C-scans. The relative translations and rotations between two C-scans are determined by carrying out a 3D cross correlation, the optimal translations in *x* and *y* directions and the rotation angle being determined from the maximum correlation coefficient.

**Fig. 9:**
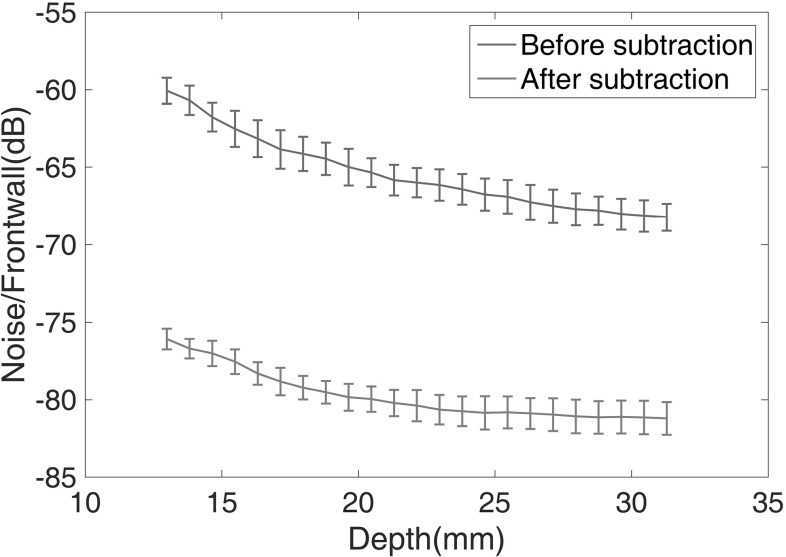
Noise to frontwall amplitude ratio versus depth before and after baseline subtraction. The data consists of ten A-scans from the first C-scan representing the baselines and the other ten traces from the second C-scan used for subtraction. Each data point shows the mean (*line*) and standard deviation (*bar*) of the RMS for a 14 mm window centred about its indicated x-axis value. The range of depths considered here corresponds to the grain noise which lies in-between the frontwall and backwall signals. Electrical noise residual is around 84 dB below the frontwall signal (Color figure online)

3D cross correlation was used to align C-scans obtained from the repeat experiments. C-scans of the backwall echo amplitude across the whole sample were carried out four times, and before each scan, the rig was recalibrated by removing and readjusting the transducer and sample. Figure 8 shows overlapped images of the first and second C-scans before and after alignment. It can be seen that before registration, the position and orientation of the sample in the two images are different; after registration, the scans are well aligned. A-scans at ten randomly distributed positions on the sample were extracted from the registered C-scans. The A-scans of the first C-scan were used as the baselines and the A-scans from the subsequent C-scans were subtracted from them. The RMS grain noise to frontwall amplitude ratio over the thickness of the sample before and after baseline subtraction was calculated for each of the ten points. The average of the grain noise level at the ten points in the second C-scan is shown in Fig. 9, together with the residual after subtraction from the corresponding signals in the first C-scan. It can be seen that after subtraction, the residual is an average of around 15 dB lower than the grain noise and there is no obvious change in the variability. Similar results were obtained with signals from the third and fourth C-scans.

It can be seen from Fig. 8 that the sample boundary is a very clear structural feature, and features like this are likely to dominate the cross correlation process. However, in some circumstance scans may be recorded over regions where there is no clear boundary. In order to test whether the registration procedure is still effective in this case, another C-scan of a small area containing no boundary of the sample was conducted. This C-scan was aligned with the first C-scan of the whole sample using the registration method. It was found that the registration process correctly located the scan of the small region onto the larger scan, showing that for highly scattering materials, grain features are sufficiently rich for satisfactory C-scan registration. After registration, A-scans at six randomly distributed positions on the sample were extracted from the C-scans. By subtracting the A-scans of the smaller area C-scan from the corresponding signals of the whole sample C-scan, an average noise reduction of around 15 dB was obtained. The results therefore show that the 3D cross correlation enables C-scans to be registered accurately enough to give residual noise levels after subtraction similar to those obtained in repeat tests at the same point.

## Compensation

As mentioned above, setup errors can cause the ultrasound wave to interact with different grains and so generate different grain noise. However, even if the setup is identical, the grain noise can still be different, since it can also be affected by other factors, such as temperature variations and transducer frequency response changes, both of which are very likely to happen in repeat scans. Temperature variations can change the wave speed, and transducer frequency response changes can affect the waveform, thereby modifying the ultrasonic response, which makes raw subtraction ineffective. Therefore, compensation for these changes need to be conducted before using baseline subtraction. In this section, compensation methods for the temperature and transducer frequency response change are presented, along with experimental results to test their effectiveness.

### Temperature Compensation

#### Influence of Temperature on Baseline Subtraction

Temperature changes can significantly influence wave propagation. Before introducing compensation methods, the influence of temperature change on baseline subtraction is briefly presented here [16].

Two tonebursts (Hanning windowed here, but the analysis is not limited to this case) $I_0$ and $I_1$, with a temperature difference $\delta T$ which causes an arrival time difference $\delta t$, are used as the baseline and current signals.

1 $$\begin{aligned} I_0&=u_0 h(t)\sin (\omega t) \end{aligned}$$

2 $$\begin{aligned} I_1&= u_0 h(t+\delta t)\sin (\omega (t+\delta t)) \end{aligned}$$

where $u_0$ is the amplitude, and in this case, *h*(*t*) is the Hanning window function, *t* is time and $\omega$ is the angular frequency.

For RF subtraction, the main concern is the phase shift caused by the temperature change, which is not affected by the window, thus the difference between *h*(*t*) and $h(t+\delta t)$ is neglected. Hence, the result of subtracting $I_0$ from $I_1$ can be written as:

3 $$\begin{aligned} I_1-I_0 \approx u_0 h(t)(\sin (\omega (t+\delta t))-\sin (\omega t)) \end{aligned}$$

Assuming $\delta t$ is small enough, the small angle approximation can be applied:

4 $$\begin{aligned} \left| I_1-I_0 \right| _\text {max}=2\pi fu_0 h(t)\delta t \end{aligned}$$

where *f* is frequency.

According to [14], the time shift due to the temperature change $\delta T$ can be expressed as:

5 $$\begin{aligned} \delta t=\frac{d}{v}\left( \alpha -\frac{k}{v}\right) \delta T \end{aligned}$$

where *d* is the propagation distance, *v* is the phase velocity, $\alpha$ is the thermal expansion coefficient and $k= \delta v/\delta T$.

Therefore,

6 $$\begin{aligned} \left| I_1-I_0 \right| _\text {max}=2\pi fu_0 h(t)\frac{d}{v}\left( \alpha -\frac{k}{v}\right) \delta T \end{aligned}$$

From Eq. (6), it can be seen that the maximum of the residual from direct subtraction increases with the temperature change, which means that the grain noise will not be suppressed effectively by direct subtraction if $\delta T$ is significant.

#### Temperature Compensation Methods

The Optimal Baseline Subtraction (or the Baseline Selection) [14, 17] has been proposed as an effective method to overcome this problem. This method establishes a database of baseline signals obtained from the undamaged structure under the different environmental conditions under which subsequent tests might be conducted. The baseline signal which is most similar to the current signal is then selected in subsequent inspections; this is generally the signal acquired at the closest temperature to that of the current test. The selection is generally done by subtracting all the baseline signals in the database from the signal collected during the inspection and choosing the one giving the minimum residual after subtraction. However, it is impractical to have a database covering all environmental conditions at a small enough temperature step.

The Optimal Stretch method is another common technique [18, 19], which uses a signal processing method to compensate for the temperature difference. As shown in Eq. (5), the temperature change introduces a time shift, which can be corrected by stretching the signal in either the time [18] or frequency [19] domain. The advantage of the Optimal Stretch method is that it only needs one baseline signal; however the drawback of the method is that the stretching distorts the waveforms as well as changing the arrival time and this, coupled with other factors such as multiple overlapping echoes, limits the temperature difference that can be compensated [16]. A combination of Optimal Baseline Subtraction and Optimal Stretch has been shown to be an effective way of reducing the number of baselines needed in the optimal baseline method while not requiring excessive stretch.

The Optimal Baseline and the Optimal Stretch techniques have been successfully applied in guided wave monitoring where the wave propagates in only one medium. However, in immersion ultrasonic testing the wave propagates in both the testpiece and the coupling medium that have very different temperature coefficients. They must therefore be considered separately.

In this paper, the Optimal Stretch technique is used to compensate for temperature changes in the sample. Since the effects of temperature on wave propagation in water and metal need to be considered separately, a two-step temperature compensation method is used. The first step is to align the frontwalls of the baseline and current signals in order to eliminate the temperature influences on the wave propagation in water; the alignment is done by cross correlating the frontwall signals. Then, a scale-transform-based stretch method [15] is used to compensate for the wave speed change in the metal.

#### Experimental Results

In order to test the effectiveness of the temperature compensation method, it was used to compensate for the difference in signals collected at different temperatures. This temperature difference was achieved by changing the temperature of the water used in the immersion tests. The water was heated up to around 33 $^\circ$C and then naturally cooled down to room temperature (around 23 $^\circ$C); a Pico PT-104 Platinum Resistance Data Logger was used to record the water temperature data. A-scans were recorded with Transducer 2 at the same position every 80 seconds; this gave 331 readings between 33 $^\circ$C and room temperature.

**Fig. 10:**
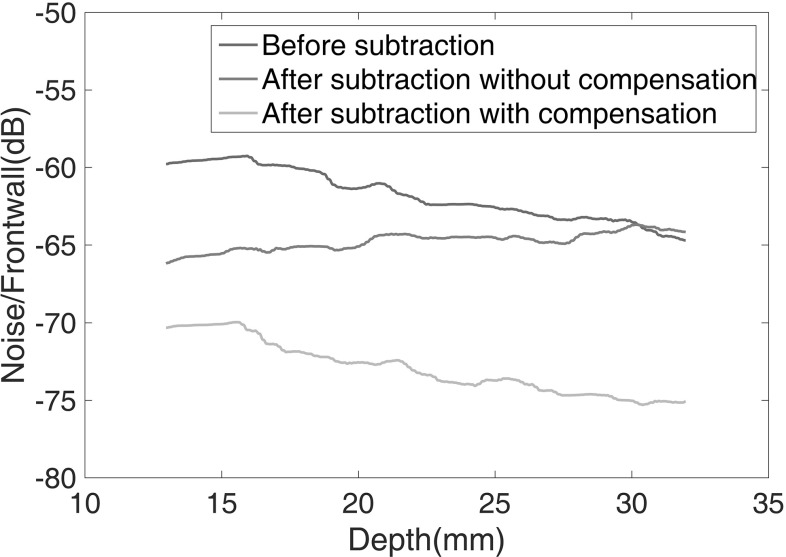
Noise to frontwall amplitude ratio versus depth before subtraction and after subtraction with and without temperature compensation. Each data point shows the RMS for a 14 mm window centred about its indicated x-axis value. The range of depths considered here corresponds to the grain noise which lies in-between the frontwall and backwall signals. The temperature of the baseline and current signal is 23 and 33 $^\circ$C respectively. Electrical noise residual is around 98 dB below the frontwall signal (Color figure online)

The noise level across the thickness of the sample at 33 $^\circ$C is shown in Fig. 10, together with the residual after subtraction from the signal at 23 $^\circ$C with and without temperature compensation. It can be seen that after subtraction without temperature compensation, the residual increases with increasing depth; at above around 30 mm, it is even higher than the original noise level. This is because the temperature change causes a wave speed change and the phase shift produced increases with distance travelled. After temperature compensation, the residual is reduced by about 11 dB compared to the original grain noise, independent of depth. This shows that temperature compensation is very effective and compared to the raw subtraction without temperature compensation, the noise reduction increases with increasing depth.

**Fig. 11:**
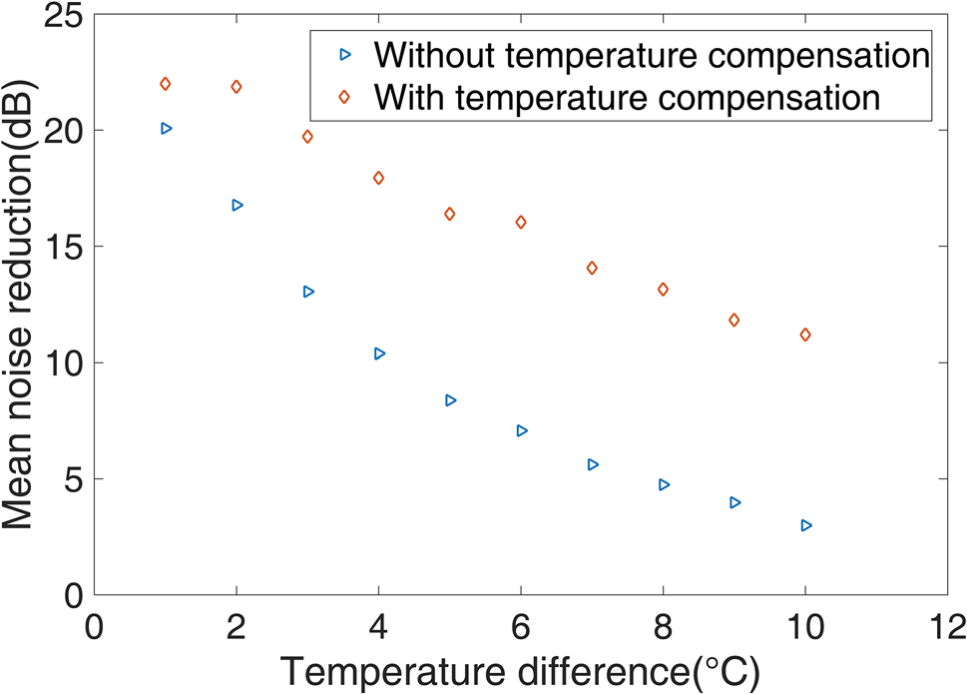
Mean noise reduction after subtraction with and without temperature compensation at different temperatures. The temperature of baseline is 23 $^\circ$C

The effect of temperature difference between the baseline and current signals was then investigated. Figure 11 shows the average noise reduction over the depth of the sample as a function of temperature difference between the current signal and the baseline at 23 $^\circ$C. The reduction in the residual noise level decreases with increasing temperature difference both with and without compensation, but the drop is significantly less rapid when compensation is applied. When the temperature difference is 1 $^\circ$C, the improvement with compensation is only 2 dB; this is because the noise reduction of about 20 dB is already at a similar level to that shown in Fig. 7 for subtraction with minimal temperature difference. At higher temperature differences the improvement rises to about 8 dB. It is shown that it is feasible to obtain a noise reduction of around 11 dB via temperature compensation and subtraction, where a temperature difference up to 10 $^\circ$C can be tolerated.

### Compensation for Transducer Frequency Response Change

In repeat inspections at long time intervals, it is likely that different transducers with similar specifications would be used in successive tests, and even if the same transducers are used, their frequency response could change due to aging. As a result, the later received signals will not have the same grain noise even though the same grains are inspected, and baseline subtraction will not perfectly remove grain noise to reveal small reflections from defects. Therefore, we need to compensate for the potential differences in transducer response. In this section, we demonstrate a frequency compensation scheme that uses the frontwall reflection to calibrate for changes in transducer frequency response, and to reduce the difference in the received grain noise so that it can be removed by baseline subtraction. This method also accounts for any change in the excitation and reception electronics. It is possible that other changes may occur due to transducer ageing, e.g., the effective transducer area could change and so alter the field pattern. However, we believe that frequency response changes are likely to be the major effect.

**Fig. 12:**
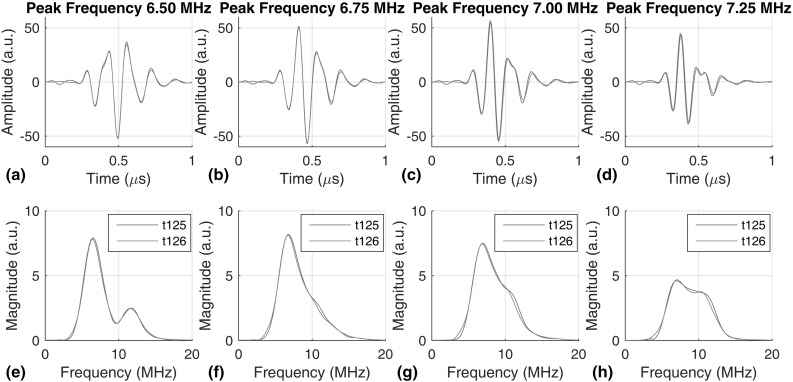
Typical A-scans showing the frontwall reflection. Plots **a**–**d** and **e**–**h** represent the time and frequency domain signals collected with different excitations, whose peak frequencies are respectively 6.5, 6.75, 7.0, and 7.25 MHz. *Blue* and *red* represent signals obtained from two different transducers (Color figure online)

#### Methodology

In an immersion inspection configuration, the ultrasonic wave excited by the transducer first propagates through a liquid medium before arriving at the interface of the testpiece. Depending on the impedance difference, a portion of the signal is reflected from the front surface and is received by the transducer, the rest of the signal being transmitted into the material. The reflected and transmitted signals have the same frequency content, and are equally affected by the change in the transducer response. Assuming linearity, the received signal *y*(*t*) can be written as the convolution of the transmitted signal *x*(*t*) with a transfer function *h*(*t*); if all other factors (inspection location, incident angle, etc) are the same, the transfer function *h*(*t*) remains the same for that particular path. If the input signal *x*(*t*) changes to a new input signal $x'(t)$, due to transducer aging or the use of a different transducer, the new received signal $y'(t)=x'(t)*h(t)$ would be different from the original *y*(*t*), limiting the utility of direct subtraction.

To reliably compare the received signals from different inspections, this difference has to be compensated, such that $y'(t)$ is similar to *y*(*t*) if no defect exists along the propagation path. Since the input signal *x*(*t*) is proportional to the frontwall reflection signal *f*(*t*), we can use the frontwall signal to calibrate the change in the transducer response and adjust the received signal in the frequency domain for both amplitude and phase differences. Considering *y*(*t*) as the baseline and $y'(t)$ as the current measurement, we obtain *y*(*t*) after compensating $y'(t)$ by:

7 $$\begin{aligned} \bar{Y}(\omega ) = F(\omega )/F'(\omega )*Y'(\omega ) \end{aligned}$$

where *F*,$F'$, and $Y'$ are the Fourier transform of, respectively, *f*,$f'$, and $y'$. To avoid extremes in the compensation term (F/F), we filtered the frequency response *F* with a threshold 15 dB below the peak of the response, i.e. we set the values below the threshold to be zero. The threshold to use in practice should be determined by the SNR of the front-wall signal. The inverse Fourier transform of $\bar{Y}(\omega )$ then resembles the baseline signal *y*(*t*), whereas any difference between the two may suggest the existence of a potential defect.

#### Experimental Setup

We test the scheme for three types of change: 1. change in the excitation frequency spectrum, 2. change of transducer, and 3. a combination of the two. Two nominally identical 7.5 MHz transducers (Transducers 2 and 3) were used to scan our specimen (shown in Fig. 3) at $41\times 16$ locations covering a $20\times 7.5$ mm area. We repeated the C-scan with each transducer four times, each time exciting the transducer with a square wave having a different pulse-width, effectively changing the frequency spectrum of the excitation. When the transducer was changed for the second set of four C-scans, the stand-off and angle adjustment techniques and the C-scan registration procedure described in Sect. 3 were used.

Figure 12 shows typical frontwall reflection signals obtained at one location. Figure 12a–d show time traces of the frontwall reflection, and Fig. 12e–h show the corresponding frequency responses. We use the peak frequencies to refer to the excitations in the following text, but note that the entire frequency spectrum changes besides the small shift in the peak frequency; the peak frequencies of the excitation in Fig. 12a–d are, respectively, 6.50, 6.75, 7.00, 7.25 MHz. The frequency responses of Fig. 12f and g are relatively simple because the excitation is well matched to the transducer resonance, whereas the 6.5 and 7.25 MHz excitations give the distorted frequency responses of Fig. 12e and h respectively.

**Fig. 13:**
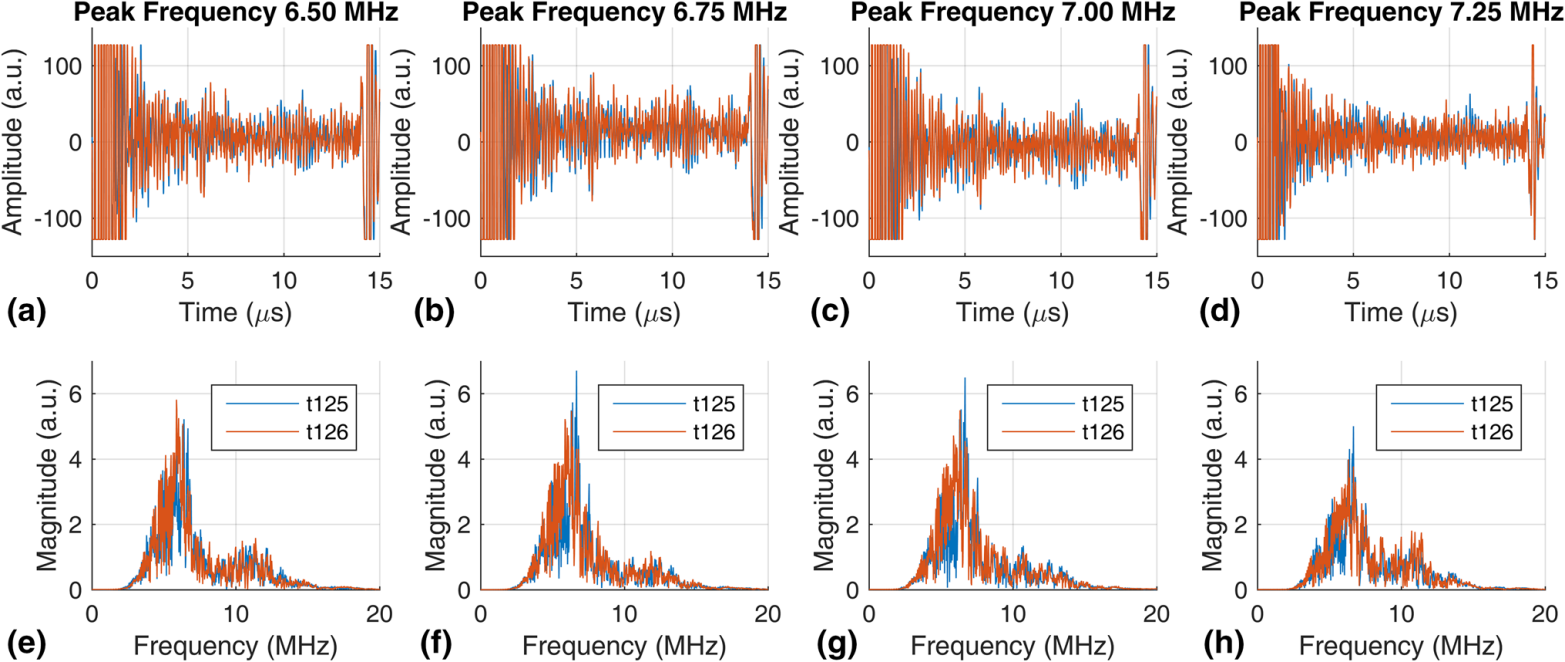
Typical A-scans showing the grain noise signals in between frontwall and backwall reflections. Plots **a**–**d** and **e**–**h** represent the time and frequency domain signals collected with different excitation, whose peak frequencies are respectively 6.5, 6.75, 7.0, and 7.25 MHz. *Blue* and *red* represent signals obtained from two different transducers (Color figure online)

It is clear from Fig. 12 that the signals from two transducers differ slightly. However, as the wave propagates through the grains, the scattered grain noise signals become substantially different, as shown in Fig. 13. Note that the frontwall (at time zero) and backwall (at 14 $\upmu$s) reflection signals in Fig. 13 are clipped because the signals are amplified to give better resolution of the grain noise signals in between.

Direct subtraction between any pair of the signals of Fig. 13 will not perfectly remove the grain noise, thereby reducing the ability to detect small defects in the material. The frequency compensation scheme aims to correct this. By comparing different pairs of signals, we simulate different practical scenarios:

I Comparing two signals from different plots with the same color simulates repeat inspections with the same transducer but with different excitation electronics or after transducer aging.
II Comparing the two signals with different colors in any plot in Fig. 13 simulates repeat inspection with different transducers but identical excitations.
III Comparing two signals from different plots with different colors simulates repeat inspection with different transducers and different excitations.

#### Experimental Results Before and After Compensation

**Fig. 14:**
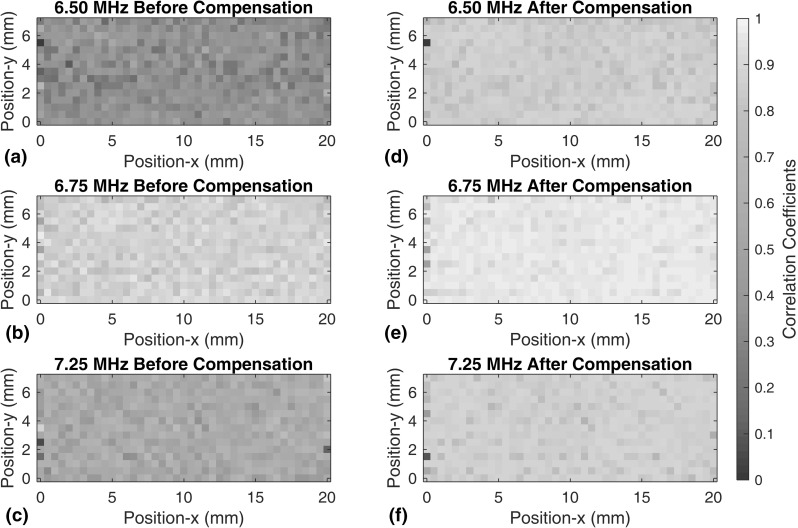
Correlation coefficients before (**a**–**c**) and after (**d**–**f**) frequency compensation at $41\times 16$ locations with an area of $20\times 7.5$ mm on the sample. Compensation is conducted using signals with peak frequency of 7.00 MHz as the baselines. (**a**, **d**), (**b**, **e**) and (**c**, **f**) correspond to the results using signals with peak frequencies of, respectively, 6.50, 6.75, and 7.25 MHz (Color figure online)

We process all pairs from the eight different signals from two transducers and four excitations, and show the results before and after the frequency compensation. We first show the absolute correlation coefficients that indicate the similarity between two signals on a 0 to 1 scale, where a unity correlation coefficient suggests the two signals have identical shapes. Figure 14 shows the correlation coefficients between the baseline signal with peak frequency of 7.0 MHz and a current signal with other peak frequencies on all $41\times 16$ locations on the sample covering an area of $20\times 7.5$ mm. The signals were all collected with one transducer but with different excitation frequency spectra, corresponding to scenario I above.

Figure 14a–c show correlation coefficients between baseline and current signals without compensation. Light-yellow pixels indicate correlation coefficients close to unity, which means the baseline and current signals are alike, and dark-blue pixels indicates the two signals are dissimilar. It can be seen that a small difference in frequency spectrum can significantly change the grain noise signals. For example, when comparing signals using 7.00 MHz excitation and signals using 6.50 MHz excitation, the mean correlation coefficient across the points is as low as 0.2.

Figure 14d–f show the correlation coefficients after the signals are compensated using the procedure of Sect. 4.2.1, the baseline being the signals with 7.00 MHz excitation. There is a clear improvement in correlation coefficients, suggesting that baseline subtraction will be more effective.

**Fig. 15:**
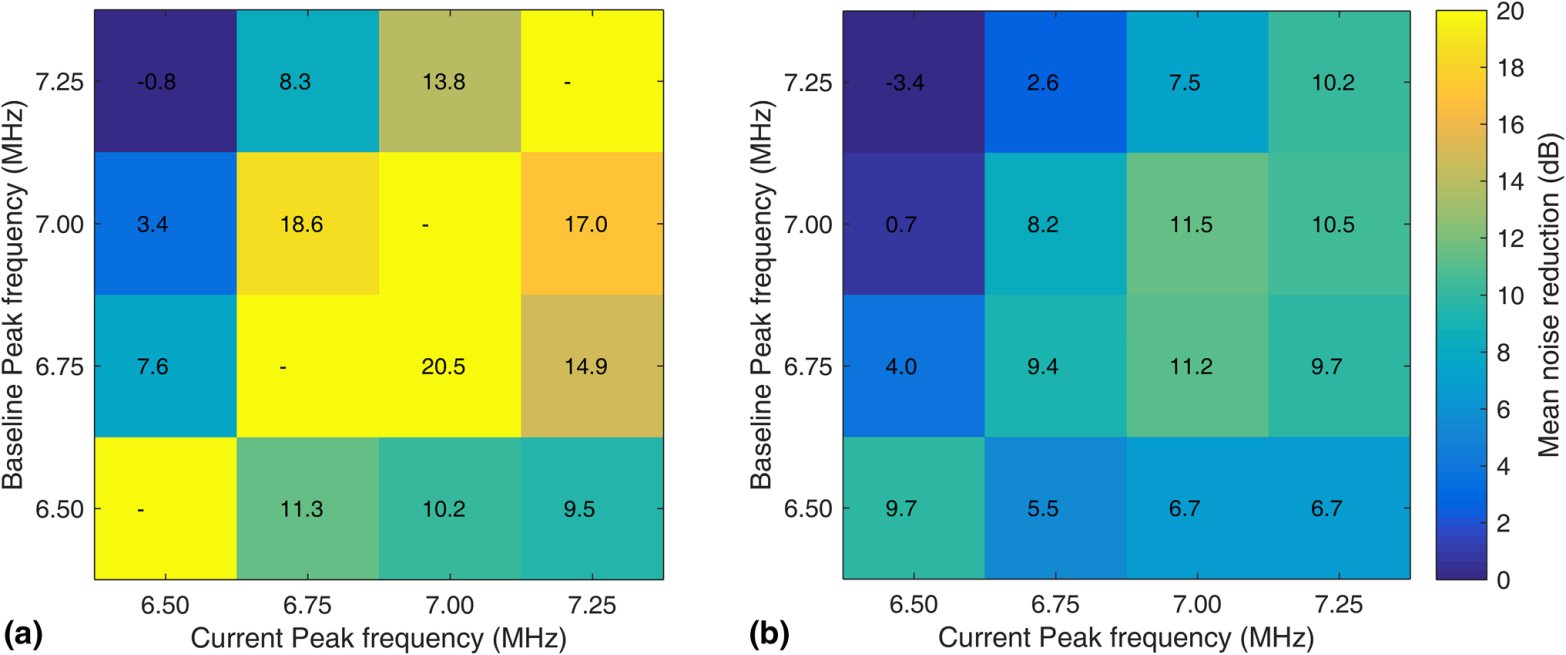
Mean noise reduction across the scan area from compensation and subtraction compared to the original baseline inspection. **a** Current and baseline signal were measured from the same transducer. **b** Current and baseline signals were measured from different transducers. Horizontal and vertical axes indicate the peak frequencies of the excitation used in, respectively, current and baseline signals (Color figure online)

Figure 15 shows the mean noise reduction across the scan area obtained by processing the signals with frequency compensation and baseline subtraction, compared to the noise level in the original baseline inspection. A *x* dB noise reduction means that by compensating and subtracting the signal, we can suppress the grain noise by *x* dB. Figure 15a shows the results where signals were taken with different excitations, where the horizontal and vertical axes correspond to the excitation peak frequencies for, respectively, the current and baseline signals used in the subtraction. As an example, the second row from the top in Fig. 15a corresponds to the test scenario in Fig. 14, where we compare current signals obtained with an excitation peak frequency of 7.00 MHz with baseline signals taken at other excitation peak frequencies. Figure 15b shows the corresponding results when the signals were taken with two different transducers.

**Table 2:** The improvement achieved by calibrating and/or compensating for various factors in baseline subtraction

Calibrated/compensated factors (differences between current and baseline signals)	Improvement level (dB)
Re-setup	
Angle	18
Stand-off	17
Angle, Stand-off and Lateral positions	15
Temperature (10 $^\circ$C test temperature change between baseline and current)	11
Transducer frequency response change (with the same transducer; relatively simple frequency spectrum; a peak frequency difference of ±250 kHz from a baseline value of 7 MHz)	14–20
Transducer frequency response change (with different transducers, thus including all above factors due to transducer re-setup; relatively simple frequency spectrum; a peak frequency difference of ±250 kHz from a baseline value of 7 MHz)	10

The diagonal line in Fig. 15a when the baseline and current excitations signals are the same is left blank because here the signals are identical. The off-diagonal values correspond to the cases where excitations are different and need to be compensated. Note that the image is asymmetric, the values in the bottom triangle being larger than their counterparts in the upper triangle. This is because the excitation was controlled by the pulse width of a square wave, so a higher peak frequency corresponds to a wider bandwidth; compensating for frequency response differences is easier when going from a wide band signal to a narrower band signal than vice versa. When compensating from wider band to narrower band for the same transducer, an improvement of 14–20 dB was obtained when the spectrum of the signal is relatively simple as in Fig. 12f and g; the gain was much smaller when the distorted spectrum of Fig. 12e was used.

The diagonal line in Fig. 15b represents the case where the baseline and current signals were taken with different transducers but with nominally the same excitations. In this case, a gain of about 10 dB was obtained from the compensation and subtraction compared to the baseline inspection. The off-diagonal values suggest that when the response spectrum has a simple form and the frequency difference between current and baseline excitations is small (where the peak frequency response changes as much as 250 kHz), a gain of over 10 dB can still be obtained from the compensation procedure; when difference between the spectra increases, the noise reduction drops quickly. It should be noted that the repeat scans with different transducers involve resetting the transducer. Therefore, the results show the combined noise reduction of the frequency response compensation and the stand-off adjustment, angle calibration and image registration procedure illustrated in Sect. 3. This is what would be involved in a real case of two inspections before and after a period in service.

### Overall Influence of Different Factors

The influence of various experimental and environmental parameters after calibration and/or compensation are compared in Table 2. It shows that if the transducer and sample are reset up with no environmental changes, using appropriate calibration methods, an improvement of 15 dB can be obtained with baseline subtraction. However modest changes in test temperature and transducer frequency response are less successfully compensated, dropping the overall improvement to 10 dB. Also the tests were done in a lab environment with no changes to the sample surface due to oxidation or corrosion that would significantly affect the ultrasonic signals. It has also been assumed that no changes to grain structure affecting the ultrasonic signal occur with exposure to service environments as indicated in [11]; if this assumption is not valid in a particular application then baseline subtraction could not be used.

## Conclusions

Coarse grained materials are desirable in the next generation of power stations where high temperatures (up to 700 $^\circ$C) will be used, due to their high creep resistance. However, in ultrasonic testing, noise due to grain scattering can mask defect echoes. To enhance defect detection sensitivity, the feasibility and reliability of comparing repeat tests using baseline subtraction to suppress grain noise has been studied.

The feasibility of setting the transducer with the same stand-off and angle relative to the testpiece in repeat immersion tests has been investigated and the influence of errors due to the transducer settings has been illustrated. A 3D cross correlation has been used to register the C-scans and so to compare the same location within the different scans; the effect of errors in the registration has been investigated. The results show that the residual grain noise can be reduced by around 15 dB via baseline subtraction, which offers a much higher defect detection sensitivity.

Since repeat tests may be carried out at different temperatures and with different transducers of similar but non-identical specifications, compensation methods for temperature variations and transducer frequency response changes have been presented and their effectiveness has been tested experimentally. After subtracting a temperature compensated baseline from a current signal collected with a temperature difference of 10 $^\circ$C, an improvement of around 11 dB was obtained. A gain of around 10 dB was achieved with the proposed frequency compensation method when compensating a small frequency shift on different transducers. Overall, it is feasible to obtain a noise reduction of around 10 dB using baseline subtraction in monitoring highly scattering materials when temperature variations are below 10 $^\circ$C and the transducer peak frequency response changes by as much as ±250 kHz from a baseline value of around 7 MHz; the dominant factors restricting the improvement gained from baseline subtraction are the temperature and transducer frequency response change. However, this relatively modest improvement is obtained at significant cost in test complexity and data storage requirements. Also, this improvement was obtained in lab conditions with no sample surface or grain structure changes.
